# Use of silver-based additives for the development of antibacterial functionality in Laser Sintered polyamide 12 parts

**DOI:** 10.1038/s41598-020-57686-4

**Published:** 2020-01-21

**Authors:** Robert D. Turner, James R. Wingham, Thomas E. Paterson, Joanna Shepherd, Candice Majewski

**Affiliations:** 1 Department of Mechanical Engineering, University of Sheffield, Royal Exchange Building, 64 Garden Street, Sheffield, S1 4BJ UK; 2 School of Clinical Dentistry, University of Sheffield, 19 Claremont Crescent, Sheffield, S10 2TA UK

**Keywords:** Antimicrobials, Mechanical engineering, Materials science

## Abstract

Infectious diseases (exacerbated by antimicrobial resistance) cause death, loss of quality of life and economic burden globally. Materials with inherent antimicrobial properties offer the potential to reduce the spread of infection through transfer via surfaces or solutions, or to directly reduce microbial numbers in a host if used as implants. Additive Manufacturing (AM) techniques offer shorter supply chains, faster delivery, mass customisation and reduced unit costs, as well as highly complicated part geometries which are potentially harder to clean and sterilise. Here, we present a new approach to introducing antibacterial properties into AM, using Laser Sintering, by combining antimicrobial and base polymer powders prior to processing. We demonstrate that the mechanical properties of the resultant composite parts are similar to standard polymer parts and reveal the mode of the antibacterial activity. We show that antibacterial activity is modulated by the presence of obstructing compounds in different experimental media, which will inform appropriate use cases. We show that the material is not toxic to mammalian cells. This material could be quickly used for commercial products, and our approach could be adopted more generally to add new functionality to Laser Sintered parts.

## Introduction

The global Additive Manufacturing (AM) market has grown by an average of 26.9% annually for the last 30 years, with the overall revenue of the industry currently estimated at $9.8 billion, and aerospace, automotive and healthcare being major sectors^1^. Parts are produced in a layer-by-layer manner, directly from a Computer-Aided Design (CAD) file. This layer-by-layer approach provides key benefits through removing the need for tooling and increasing the ease with which complex geometries can be produced. However, despite their clear potential, the range of materials that can be used in AM processes is limited compared to more traditional manufacturing techniques, which in turn has restricted the range of applications in which they can be used.

Laser Sintering is an AM technology that produces parts by selectively scanning and melting consecutive cross-sections of polymer powder particles. Areas which have not been scanned remain as loose powder throughout the process, acting to support any overhanging areas, which in turn allows the economic production of highly complex part geometries. This geometric capability makes Laser Sintering highly suited to production of complex, optimised, products and devices, or to the production of products and devices personalised to individuals. However, particularly when considering hand-held and/or medical products, increasing geometric complexity can render them difficult and time-consuming to clean effectively, potentially providing increased chance of spread of bacteria. Incorporation of antibacterial properties into the parts themselves could reduce or eliminate this risk, and is the focus of this work.

Many antimicrobial products are currently available to purchase, with a growing global market for antimicrobial additives (materials that are added to base materials to yield antimicrobial properties). Antimicrobial products are used in the healthcare sector as well as in consumer goods - the demand for these is driven not only by a desire to improve health, but to develop opportunities to create added value. They have the potential to make a positive impact in healthcare in implants, prosthetics and splints^2^, and surfaces and devices in clinical settings (e.g. sinks, instruments, keyboards). Adlhart and colleagues propose a helpful set of categories for antimicrobial surfaces: anti-adhesive surfaces (based on preventing microbial attachment^3^), contact-active surfaces (that kill microbes on contact) or biocide releasing surfaces (from which the active ingredient is eluted into the surroundings)^4^. If a material is not inherently antimicrobial, the different types are obtained either by mixing antimicrobial additives into the bulk material, or through direct physical modification (e.g. altered roughness) or chemical modification (e.g. attachment of a biocide) to the surface. The biocidal properties of copper, zinc and silver (amongst other metals) are exploited in commercial antimicrobial additives and in ongoing research^5^, with silver a well-established choice. We therefore chose to develop a composite material to be used in AM using silver-based additives.

Research into AM of antimicrobial materials is a small but rapidly growing field. Researchers have incorporated antimicrobials (or antibiotics) into polymer, metal and ceramic composites using a range of AM techniques. Fused Deposition Modelling (FDM) has been used to incorporate, for example, nitrofurantoin^6^ or silver nitrate^7^ for potential use in medicine and healthcare. Stereolithography (SLA) has been used to incorporate 4-aminosalicylic acid^8^ for customised drug-release kinetics, silver nanoparticles^9^ or quaternary ammonium compounds^10^. Binder Jetting has been employed, often exploiting the capacity to dissolve antibiotics in the liquid binder, to form polymer^11^ or ceramic^12^ composites, for potential bone implants amongst other applications. Robocasting has been used to incorporate a range of agents including levofloxacin^13^, quaternary ammonium compounds^14^ or silver nanoparticles^15^, in a field again largely aimed at developing bone implants. Selective Laser Melting (SLM) of titanium^16^ or cobalt-chromium-molybdenum^17^ has been employed by several groups to develop bone implants – adding antimicrobial functionality to these generally requires a post-processing step, likely due to the high temperatures involved in melting metal being deleterious to many antimicrobials. To the best of our knowledge, no antimicrobial materials made using Laser Sintering have been described to date in the academic literature.

The proposed antimicrobial effects of silver remain an active research topic and fall into two broad categories: direct reactions between silver ions and cellular components (proteins, membranes, DNA) or indirect damage caused by reactive oxygen species (e.g. hydrogen peroxide) generated by the silver^18,19^. Xiu and coworkers showed that silver ions are equally toxic to *Escherichia coli* in aerobic and anaerobic conditions, contending on the basis of this that reactive oxygen has no role in the mechanism of action. However, an earlier study of *E. coli* and *Staphylococcus aureus*^20^ contradicts this finding, arguing that there is a role for oxidative stress under aerobic conditions. It may be that this inconsistency can explained by methodological differences. An important study highlights the protective effect of thiol groups in the experimental medium^21^. It seems reasonable on the basis of this to assume that silver can bind these thiols and as a result become unavailable to participate in any other reactions that might (directly or indirectly) harm microbes. It has also been established that increased levels of serum in growth medium for cultured human cells is protective against the toxic effects of silver^22^, perhaps by a similar thiol-binding mechanism. Whatever the mechanism of antimicrobial action, our concern as engineers is to develop an approach that can safely kill or inhibit microbes using silver ions, and all relevant previous research suggests that to do this silver ions must be available in proximity to the microbes. In this manuscript, we do not contribute to the debate on the mechanism of action of silver as an antimicrobial, but explain how our material delivers silver ions and under what circumstances it will be most useful.

Here, we have adopted a commercially available antimicrobial additive (Biocote B65003) and combined this with a widely used Laser Sintering powder (polyamide 12, EOS PA2200) to create an antimicrobial material suitable for a range of potential uses. We characterise the resulting composite parts, including their mechanical properties, antibacterial properties (using *S. aureus* as a model Gram positive organism and *Pseudomonas aeruginosa* as a model Gram negative), and demonstrate a lack of cytotoxic effects.

## Results and Discussion

### Processability of material

**Figure 1:**
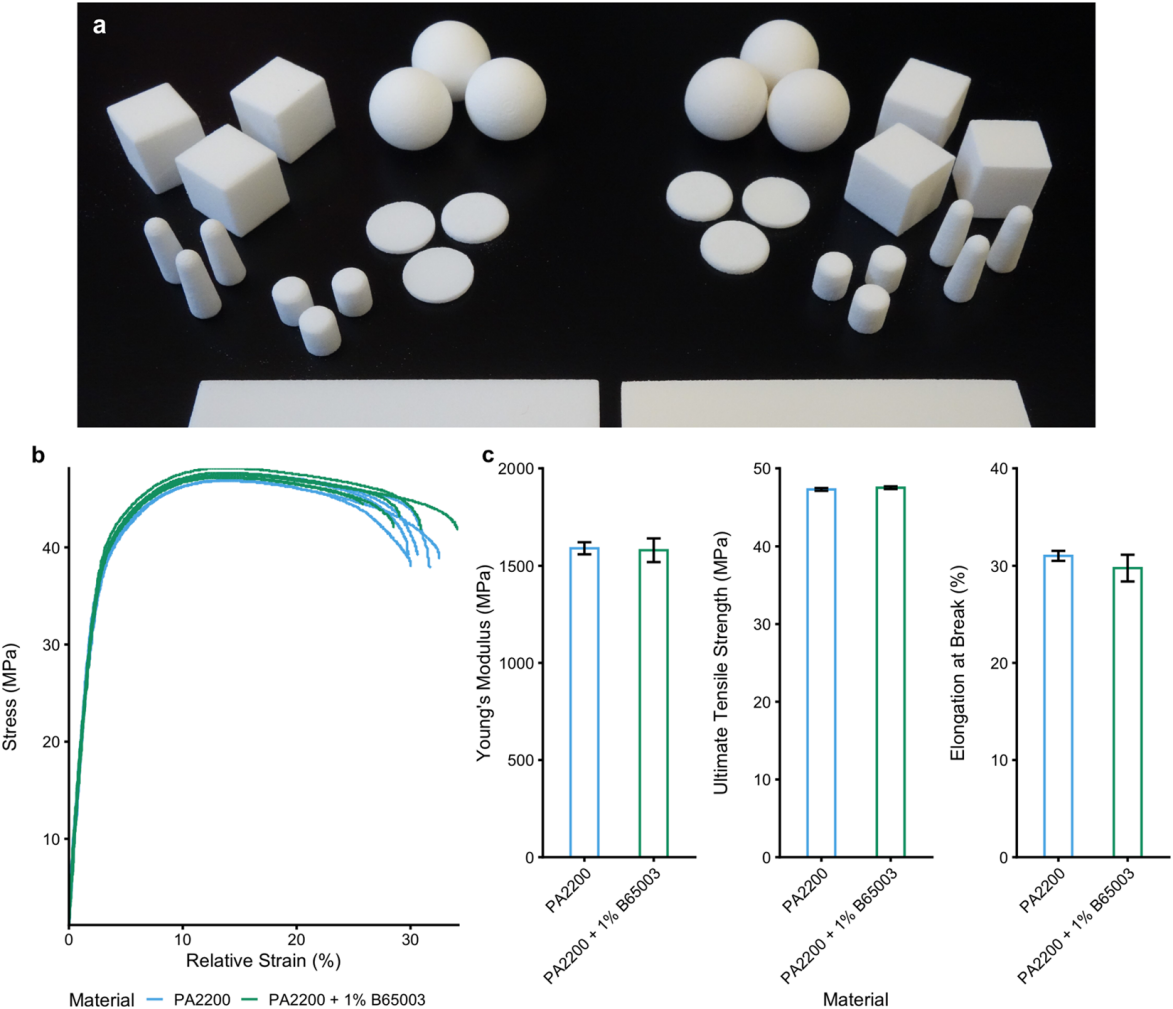
Engineering properties of parts (**a**) Photograph of a selection of parts made from PA2200 (left) alongside the 1% B65003 composite material (right). (**b**) Raw stress-strain curves from tensile testing. (**c**) A comparison of Young’s modulus (*E*), ultimate tensile strength (*σ*_uts_) and elongation at break (*ε*_max_) for both materials.

We printed polyamide 12 parts in a range of geometries using standard settings for this material on a commercial EOS Formiga P100 printer. We subsequently printed the same part geometries in polyamide 12 mixed with 1% B65003 silver phosphate glass. The parts were qualitatively near-identical (Fig. 1a). This straightforward experiment demonstrated the processability of a mix of sinterable and non-sinterable powders via a Laser Sintering 3D printer.

### Mechanical properties of parts

Tensile testing was employed to ensure that there were no detrimental effects on the mechanical properties of the composite when compared with the standard polyamide 12 Laser Sintering material. We therefore printed “dogbones” for use in tensile testing, which involved measuring the force required to break the parts when subjected to a tensile load. This yielded measurements of Young’s Modulus (*E* – the stiffness of the material), Ultimate Tensile Strength (*σ*_uts_ – how much force per unit area is needed to break the material) and Elongation at Break (*ε*_max_ – how much the material ‘stretches’ before breaking) which were similar for both materials (Fig. 1b,c): The properties of the Laser Sintered parts were measured as; polyamide 12 (*E* = 1590 ± 69 MPa, *σ*_uts_ = 47.3 ± 0.4 MPa, *ε*_max_ = 31.0 ± 1.2%) and 1.0% B65003 (*E* = 1580 ± 136 MPa, *σ*_uts_ = 47.5 ± 0.4 MPa, *ε*_max_ = 29.8 ± 3:1%). A 2 sample Welch’s t-test was carried out to compare the materials to each other, with *p* values < 0.05 considered to be significantly different. The resulting *p* values were; 0.89 for *E*, 0.39 for *σ*_uts_, and 0.43 for *ε*_max_ showing that there was no significant difference. These results indicate the materials can be used interchangeably, with no effect on the mechanical properties of printed parts.

### Part structure and composition

**Figure 2:**
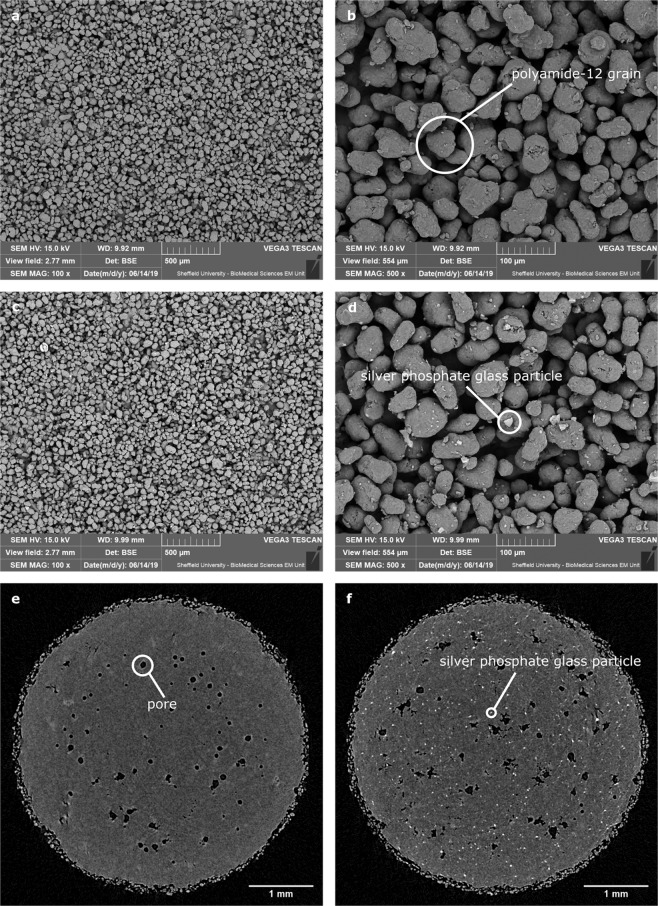
Images of parts – (**a**,**b**) SEM images of base material (sintered PA2200), (**c**,**d**) composite material. The lighter, more angular objects in d are likely silver phosphate glass. (**e**) X-Ray micro tomography section of base material (PA2200), showing pores. (**f**) X-Ray micro tomography section of the composite showing even distribution of silver phosphate glass particles.

Parts made of the base material and composite were imaged using SEM. This revealed the characteristic granular structure of the surface of the Laser Sintered polyamide (Fig. 2). Some differences in contrast were observed between the surface of the base material and the composite (compare Fig. 2a,b with Fig. 2c,d) due to the sensitivity to material of backscattered electrons.

The silver phosphate glass was found to be distributed evenly throughout the parts on this basis of X-Ray micro tomography. The difference in electron density between this and the base polyamide 12 led to the silver phosphate glass appearing brighter, allowing analysis of its dispersion (Fig. 2e,f).

**Figure 3:**
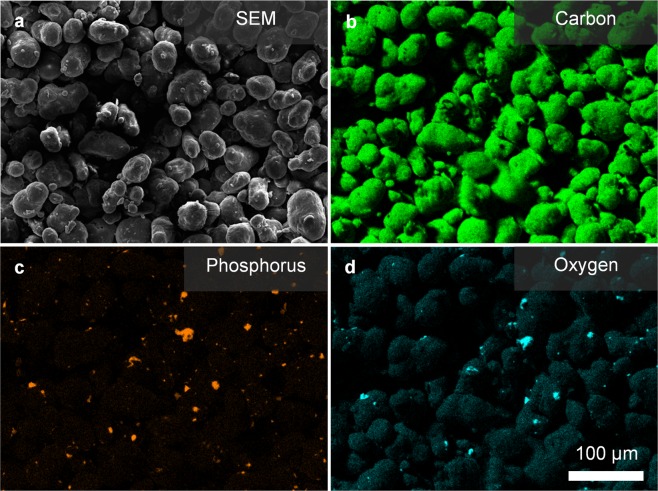
SEM and EDX map of composite PA2200 + B65003 surface. (**a**) SEM micrograph of the surface scanned with EDX. (**b**–**d**) Elemental map from EDX analysis of the surface with colour indicating detection of the corresponding element.

EDX elemental analysis was used to identify the distribution of the additive on the surface. A spectral analysis of both the B65003 additive and the composite part (Table 1), showed that although silver was present in the additive, the low concentration was such that it was below the detection limit when combined in the printed part. To map the location of the additive (Fig. 3), the elements phosphorus and oxygen were instead used as these were abundant in the additive but not in PA2200, as shown in Fig. 3c,d.

**Table 1:** Composition of the silver phosphate glass additive (B65003) and the composite part (PA2200 + 1.0% B65003) obtained from EDX analysis.

Sample	Weight percentage ± Standard Deviation (%)						
	O	C	P	Ti	Mg	Ca	Ag
B65003	63.6 ± 0.4	16.5 ± 0.4	12.6 ± 0.1	0.0 ± 0.0	3.5 ± 0.0	2.9 ± 0.0	0.9 ± 0.1
PA2200 + 1.0% B65003	72.5 ± 0.1	27.1 ± 0.1	0.2 ± 0.0	0.1 ± 0.0	0.0 ± 0.0	0.0 ± 0.0	0.0 ± 0.0

### Composite parts release silver

Having validated the engineering properties of the parts, we next determined the extent to which the active antimicrobial, silver, was eluted. This was measured using Inductively Coupled Plasma-Optical Emission Spectroscopy (ICP-OES) which detects the concentration of silver ions in solution. In a preliminary test, silver phosphate glass was found to release far more silver ions than metallic silver powder, further validating this material as an antimicrobial additive (Table 2). We estimate our parts (containing 1.0% B65003) released a similar amount of silver per unit surface area over 24 hours (~0.012 mg/l/cm^2^) as a previously reported compression moulded polyamide 12 composite containing 1.4% Nanosilver (~0.03 mg/l/cm^2^)^23^ released over 100 days. This indicates that our new AM approach yields a material that releases silver (loosely speaking) at least as well as existing (non-AM) materials. The surface area of our spheres (used in this calculation) was 4.62 ± 0.01 cm^2^ (standard error, *n* = 100 {20 parts, 5 measurements per part, pooled}).

**Table 2:** ICP-OES measurements of silver concentration.

Sample	Mean Standard Error	n samples above 0.002 mg/l detection limit (/total)
**Silver ion concentration after 24 hours incubation of silver or silver phosphate glass at 2 mg/ml in water**		
Control (Water)	NA	0/4
Silver Powder	0.006 ± 0.0016 mg/l	3/4
B65003	26 ± 2.5 mg/l	4/4
**Silver ion concentration after 24 hours incubation of polyamide 12 or antibacterial polyamide 12 (containing 1.0% B65003) spheres in 5 ml water**		
PA2200	NA	0/3
PA2200 + 1.0% B65003	0.056 ± 0.018 mg/l	3/3

### Composite parts have an antibacterial effect against gram positive *Staphylococcus aureus* and gram negative *Pseudomonas aeruginosa*

**Figure 4:**
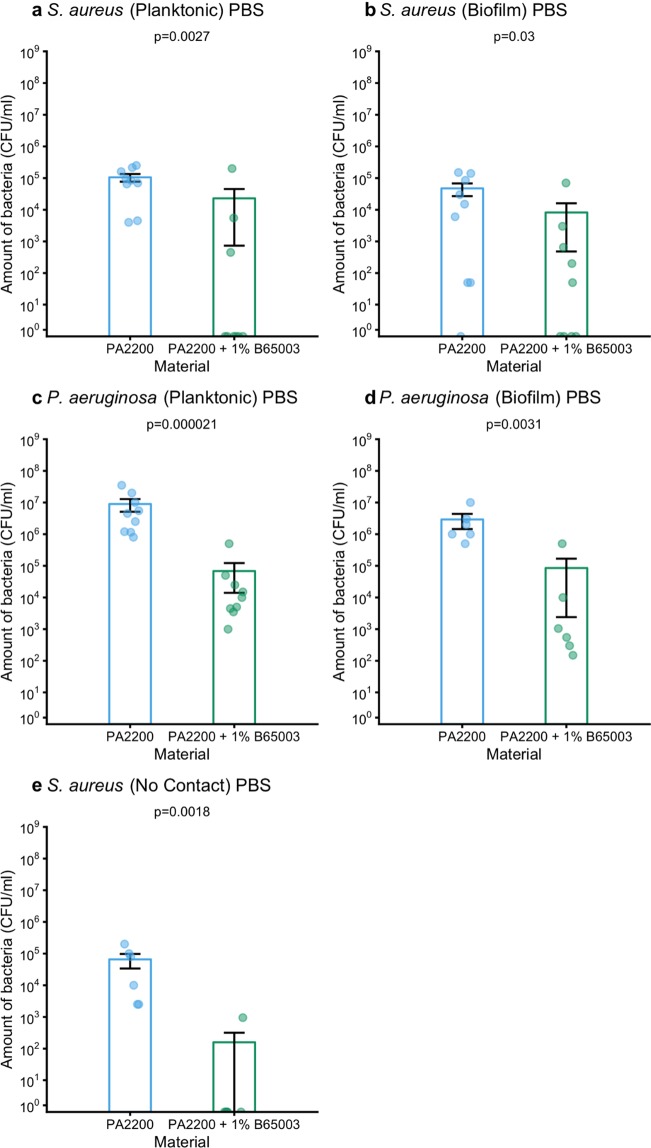
Comparison of amount of *S. aureus* (CFU/ml) in PBS surrounding (**a**), or attached to (**b**) polyamide 12 or antibacterial polyamide 12 (containing 1.0% B65003); and comparison of amount of *P. aeruginosa* (CFU/ml) in PBS surrounding (**c**), or attached to (**d**) polyamide 12 or antibacterial polyamide 12 (containing 1.0% B65003). Comparison of amount of *S. aureus* (CFU/ml) in PBS previously incubated with polyamide 12 or antibacterial polyamide 12 (containing 1.0% B65003) (**e**).

Assured that our composite parts release silver, we then checked for antibacterial activity against two representative pathogens. Parts were incubated in Phosphate Buffered Saline (PBS) containing bacteria for 24 hours, after which the amount of bacteria in the medium (planktonic bacteria) and on the parts surface (biofilm) was measured. Bacteria survived for 24 hours in PBS with a normal polyamide 12 part. There were fewer planktonic *S. aureus* in PBS that had held a part made of the antibacterial composite than in the equivalent for polyamide 12, with some samples containing so few bacteria that they were undetectable in our assay (Fig. 4a). This effect was mirrored for *S. aureus* biofilms (Fig. 4b). In the case of *P. aeruginosa* there was also a reduction in numbers of bacteria comparing antibacterial polyamide 12 (containing 1.0% B65003) to polyamide 12 for both planktonic organisms and those in biofilms on the surface of the parts (Fig. 4c,d).

### Antibacterial effects do not require contact between parts and bacteria

To explore whether the anti-biofilm properties of our material were mediated by contact between bacteria and parts, we incubated parts in PBS, removed them after 24 hours and then inoculated the PBS with bacteria. There were less bacteria in PBS that had contained antibacterial polyamide 12 (containing 1.0% B65003) than that which had held normal polyamide 12 (Fig. 4e). This proves that contact between parts and bacteria is not required for an antibacterial effect (it does not totally disprove that there may also be some contact mediated effects). We infer from this that silver ions are eluted from the antibacterial polyamide 12 (containing 1.0% B65003) in solution and diffuse to their targets. The anti-biofilm effect is therefore likely a consequence of bacterial mortality (likely prior to adhesion to the antibacterial polyamide 12 (containing 1.0% B65003)) rather than an anti-adhesion mechanism.

### Antibacterial effects are diminished in rich media

**Figure 5:**
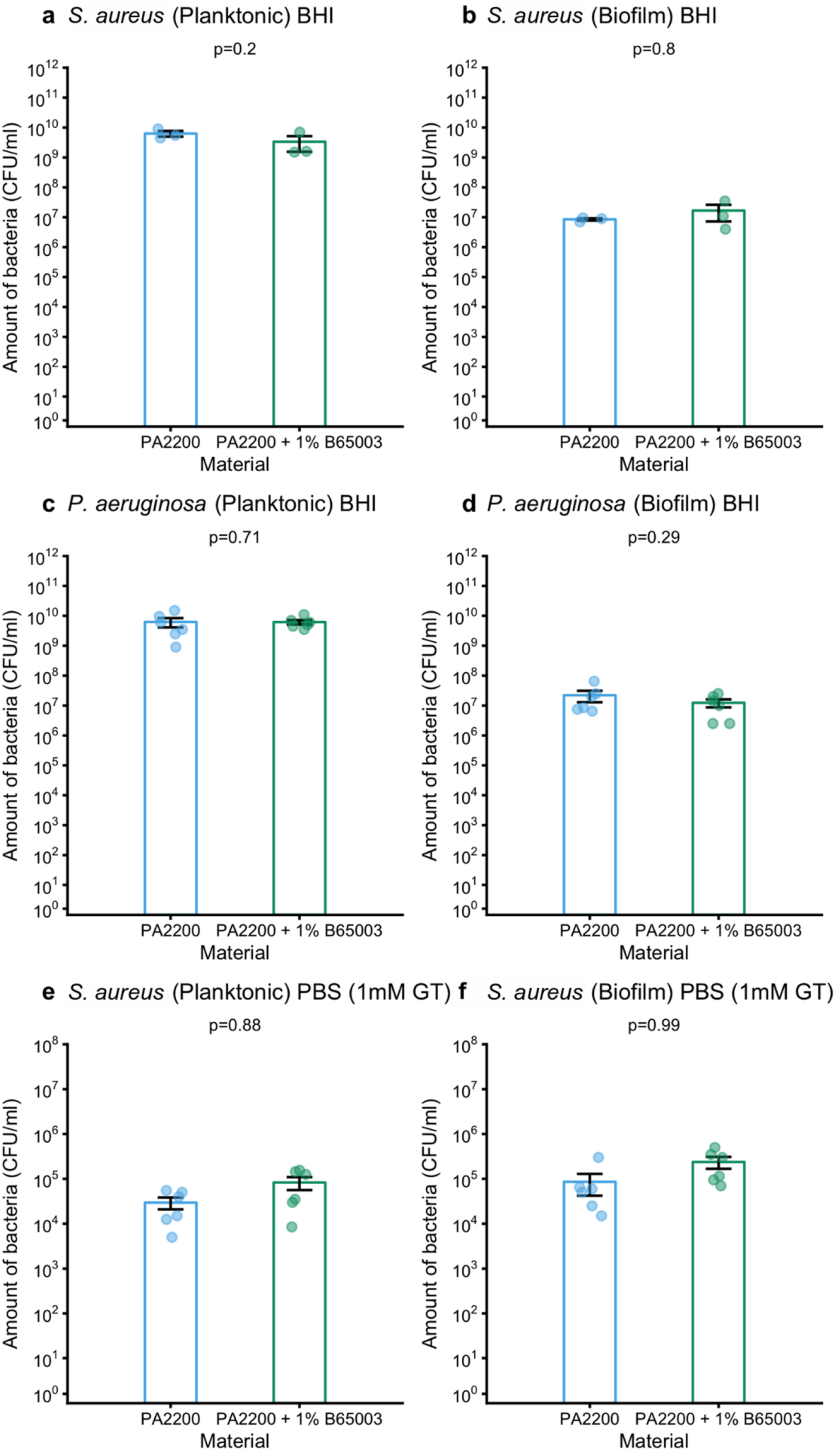
(**a**) comparison of the amount of *S. aureus* (CFU/ml) in BHI surrounding, or attached to polyamide 12 or antibacterial polyamide 12 (containing 1.0% B65003), (**b**) comparison of amount of *P. aeruginosa* (CFU/ml) in BHI surrounding, or attached to polyamide 12 or antibacterial polyamide 12 (containing 1.0% B65003), (**c**) comparison of the amount of *S. aureus* (CFU/ml) in PBS containing 1 mM reduced glutathione surrounding, or attached to polyamide 12 or antibacterial polyamide 12 (containing 1.0% B65003).

PBS is reasonably representative of a very nutrient-poor (but osmotically stable and close to pH neutral) environment for bacteria, and is most relevant to partly hydrated fomite surfaces such as one might find in a kitchen or bathroom environment, or in some clinical or semi-clinical settings. However, we were also interested in the efficacy of our parts in a rich-media environment; one containing a complex mix of biologically derived molecules in which bacteria had the nutrients needed to grow and divide. To investigate this we used Brain Heart Infusion (BHI), a commonly used rich medium for growing either *S. aureus* or *P. aeruginosa*. In this medium there was no difference between bacteria grown in media containing either polyamide 12 or antibacterial polyamide 12 (containing 1.0% B65003) for either species (Fig. 5a–d).

We hypothesised, informed by previous work^21,22^, that the difference in performance between PBS and BHI was due to the presence of chemicals in BHI that react with silver ions, rendering them unable to perform their antibacterial function, particularly molecules with thiol (sulfhydryl) functional groups. To test this, we repeated our experiments in PBS containing 1 mM reduced glutathione (Fig. 5e,f). We saw no substantial difference in the efficacy of parts made of polyamide 12 or antibacterial polyamide 12 (containing 1.0% B65003) in this medium. This result, in the context of previous findings, strongly suggests that the presence of confounding chemicals explains the relative lack of efficacy in BHI as compared with PBS.

### Composite parts do not have a cytotoxic effect

**Figure 6:**
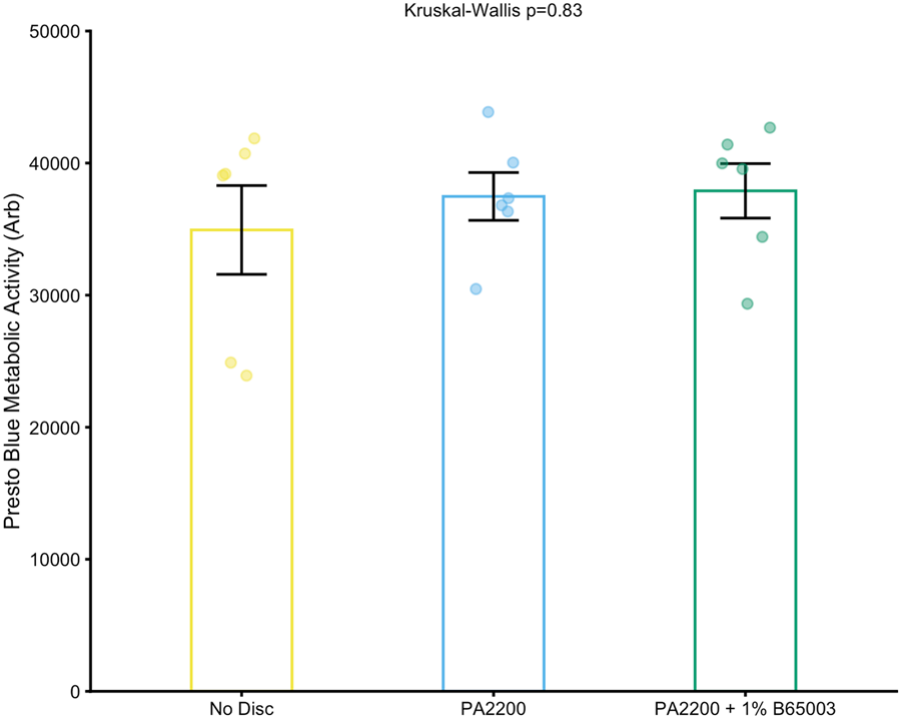
Comparison of metabolic activity between fibroblast monolayers containing parts, a disc made of polyamide 12 or one made of antibacterial polyamide 12 (containing 1.0% B65003). A Kruskal-Wallis test indicates no difference between groups.

For any use case where parts are to come into contact with humans or animals, it is important to determine levels of toxicity. This was achieved by incubating parts with 2D monolayers of human fibroblast cells at the bottom of tissue culture wells. Such cells make up connective tissue, including dermal skin layers, and produce collagen. We found no difference in metabolic activity (measured using a Presto Blue assay) between any of the wells containing either no part, a part made from polyamide or antibacterial polyamide 12 (containing 1.0% B65003) (Fig. 6).

## Conclusions

We have developed an approach for producing Additive Manufactured parts with antibacterial properties for use in the Laser Sintering process. The engineering properties of the new composite are indistinguishable from those of the standard polyamide 12 base material. The material is most effective in nutrient-poor hydrated environments (where reactions that interfere with the activity of the silver are less likely) and under these circumstances is able to reduce numbers of planktonic bacteria in its surroundings and numbers of biofilm bacteria attached to the surface. We have shown that there are no fundamental issues with cytotoxicity.

Efficacy against such diverse bacterial species as *S. aureus* and *P. aeruginosa*, in the context of the long history of silver as an antimicrobial agent, strongly suggests a spectrum of action against both Gram positive and Gram negative bacteria. Additional work would determine the breadth of antimicrobial activity of our material across other species, but given our evidence we would expect our material to be effective against further unwanted bacteria.

There are a range of potential uses for the material where favourable circumstances are present, including those that are intermittently hydrated, in kitchens, bathrooms and on hospital wards. However, a demonstrable lack of efficacy in rich BHI media and in PBS containing reduced glutathione should temper expectations of performance in nutrient-rich environments (e.g. *in vivo* or in the food industry). Future work will include field trails to validate laboratory results in real-world settings.

## Methods

### Materials

A polyamide 12 based powder was chosen as the base material (PA2200, EOS) for the antimicrobial composite. This was chosen as it is widely used in industry, meaning any added functionality could have a high impact in a relatively short timescale. A commercially available silver phosphate glass (B65003, BioCote) was chosen to create the antimicrobial effect. This material was provided in powder form, with particle size <40 μm and with an angular geometry as shown in Fig. 2d. Prior to printing, the powders were mixed for approximately 100 minutes in a rotary tumbler with 1.0% (by mass) of B65003 added to virgin (unused) PA2200.

### Laser sintering

Parts were produced using an EOS Formiga P100 with the standard settings for PA2200. The key parameters used for both materials were, laser power 21 W, scan spacing 0.25 mm, and scan speed 2500 mm/s; as this was an experimental material no additional contour scan was used. Loose powder was removed from the parts using compressed air only, to limit the potential sources of contamination.

### Mechanical properties

**Figure 7:**
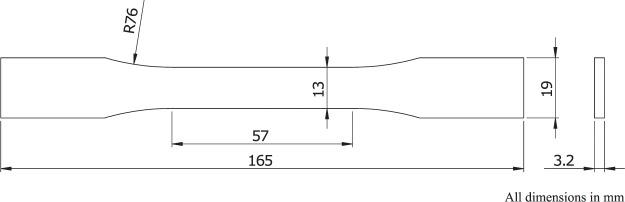
Dimensions of a type I tensile test specimen according to ASTM D638.

Tensile testing was carried out using a Tinius Olsen 5K with Laser Extensometer, in accordance with the methodology in ASTM D638. A type I specimen was used (Fig. 7), with a minimum of 5 specimens per material; the Young’s Modulus (*E*), Ultimate Tensile Strength (*σ*_uts_), and Elongation at Break (*ε*_max_) were determined to characterise the mechanical properties.

### Scanning electron microscopy

Samples were gold sputter-coated, then imaged using a TESCAN VEGA3 SEM using an accelerating voltage of 15 kV and detecting backscattered electrons.

### Energy-dispersive x-ray spectroscopy

Samples were gold sputter-coated and a TESCAN VEGA3 SEM with attached Oxford EDX analysis was used. EDX whole area mapping using secondary electron at 15 kV with elements detected automatically chosen by the software (AZtec, Oxford instruments) based on all elements detectable within the sample.

### Micro-CT

5 × 5 mm cylinders were built and scanned using a Skyscan 1172 MicroCT Scanner using the following parameters: pixel size: 4.87 μm, voltage: 40 kV, intensity: 144 μA, total rotation: 180°, rotation step: 0.35°, time: 18 minutes, filter: none.

### Inductively coupled plasma – optical emission spectroscopy

Powders or parts were incubated for 24 hours at 37 °C with agitation in distilled water. Supernatant was extracted and, for powder samples, was centrifuged (15 minutes at 2000 rpm) and filtered (0.2 μm filter) to remove suspended powder particles. ICP-OES was conducted using a Spectro-Ciros-Vision Optical Emission Spectrometer.

### Bacterial strains and growth conditions

Bacterial strains used were clinical isolates of *Staphylococcus aureus* (S235) and *Pseudomonas aeruginosa* (SOM1) from the Charles Clifford Dental Hospital, Sheffield^24^. These were maintained on Brain Heart Infusion (BHI) agar (Sigma-Aldrich) plates at 37 °C. Liquid cultures were in BHI broth (Oxoid). Species of bacteria come in a variety of “strains”. We chose to use “clinical isolates” (strains taken from infected patients) as opposed to strains sourced from a standard strain collection, as clinical isolates are more representative of the types of bacteria that are encountered in the real environment. BHI is a widely used medium derived from animal products it has all the nutrients that the bacteria need. As both *P. aeruginosa* and *S. aureus* are capable of living in a human host, we grew them at body temperature, 37 °C.

### Incubation of parts with bacteria

Overnight cultures were diluted to OD_600_ = 0.01 (this is a proxy measurement for the number of bacteria in a liquid a – typical maximum is 10) in either PBS or BHI. This solution (5 ml) was then added to glass universals containing autoclaved test parts and incubated with agitation (so all of the test part was equally exposed) at 37 °C for 24 hours.

### Release of biofilms

Parts were washed twice in PBS to release poorly attached bacteria before being vortexed for 30 s in 2 ml PBS to release the well attached biofilm.

### Quantification of colony forming units

Bacterial suspensions were serially ten-fold diluted 10:90 μl in PBS in 96-well microtitre plates. Each well in the plate then contained a number of bacteria that could be related back to the parent concentration. Subsequently, 20 μl of each dilution was spotted onto BHI agar, such that at sufficiently high dilutions each individual bacterium could form a colony distinguishable from its neighbours, before overnight incubation and colony counting. Once bacteria had been counted at the dilution where the highest density of colonies could be distinguished, we were then able to calculate the number of bacteria in the parent culture using Eq. 1.

1 $${\rm{CFU}}/{\rm{ml}}={\rm{Count}}\times {10}^{{\rm{dilution}}}\times 50$$

The term Colony Forming Units (CFU) is used, as with this method we determine the number of colonies, not individual bacteria and in some cases more than one bacterium may be aggregated together in a single CFU.

### Eukaryotic cell culture conditions

To obtain human dermal fibroblasts, human skin was obtained anonymously for medical research, with written informed consent, from individuals undergoing elective surgery for breast reduction or abdominoplasty. All methods were carried out in accordance with relevant guidelines and regulations and all experimental protocols were approved by the NHS Research Ethics Committee (REC), reference: 15/YH/017.

To isolate primary fibroblasts, dermis from donated skin was minced, then digested by 0.05% collagenase A in Dulbecco’s Modified Eagle’s Medium (DMEM) overnight at 37 °C with 5% CO_2_. The cell suspension was centrifuged at 400 g and the pellet resuspended in medium (DMEM supplemented with 10% fetal calf serum (FCS), 0.25 mg.mL^−1^ glutamine, 0.625 μg.mL^−1^ amphotericin B, 100 I.U.mL^−1^ penicillin, and 100 μg.mL^−1^ streptomycin). Cells were cultured in fibroblast medium in T25 flasks and incubated at 37 °C with 5% CO_2_. Medium was changed every 2 days, and cells were passaged as needed.

### Quantification of metabolic activity in eukaryotic cells

Fibroblasts were seeded at 50,000 cells/ml in 12 well plates (1 ml/well) and cultured for 24 hours before parts were added. After a further 24 hours parts were removed and growth media was replaced with the same containing 10% Presto Blue (Invitrogen). This was allowed to develop for 40 minutes before readout on a Tecan Infinite 200 plate reader in fluorescence mode with excitation at 535 nm and emission at 590 nm. The fluorescence of the Presto Blue is proportional to the metabolic activity of the cells.

## Data Availability

The datasets generated during and/or analysed during the current study are available from the corresponding authors on reasonable request.
